# CASCADE: a phase 2, randomized, double-blind, placebo-controlled, parallel-group trial to evaluate the effect of tezepelumab on airway inflammation in patients with uncontrolled asthma

**DOI:** 10.1186/s12931-020-01513-x

**Published:** 2020-10-13

**Authors:** Claire Emson, Sarah Diver, Latifa Chachi, Ayman Megally, Cherrie Small, John Downie, Jane R. Parnes, Karin Bowen, Gene Colice, Chris E. Brightling

**Affiliations:** 1 Translational Science and Experimental Medicine, Research & Early Development, Respiratory & Immunology, BioPharmaceuticals R&D, AstraZeneca, Gaithersburg, MD USA; 2 University of Leicester, Leicester, UK; 3 Respiratory & Immunology, BioPharmaceuticals R&D, AstraZeneca, Gaithersburg, MD USA; 4 Development Operations, BioPharmaceuticals R&D, AstraZeneca, Mississauga, Ontario Canada; 5 Amgen, Thousand Oaks, CA USA; 6 Biometrics, Respiratory & Immunology, BioPharmaceuticals R&D, AstraZeneca, Gaithersburg, MD USA

**Keywords:** Asthma, Tezepelumab, T2 inflammation, TSLP

## Abstract

**Background:**

Patients with severe, uncontrolled asthma, particularly those with a non-eosinophilic phenotype, have a great unmet need for new treatments that act on a broad range of inflammatory pathways in the airway. Tezepelumab is a human monoclonal antibody that blocks the activity of thymic stromal lymphopoietin, an epithelial cytokine. In the PATHWAY phase 2b study (NCT02054130), tezepelumab reduced exacerbations by up to 71% in adults with severe, uncontrolled asthma, irrespective of baseline eosinophilic inflammatory status. This article reports the design and objectives of the phase 2 CASCADE study.

**Methods:**

CASCADE is an ongoing exploratory, phase 2, randomized, double-blind, placebo-controlled, parallel-group study aiming to assess the anti-inflammatory effects of tezepelumab 210 mg administered subcutaneously every 4 weeks for 28 weeks in adults aged 18–75 years with uncontrolled, moderate-to-severe asthma. The primary endpoint is the change from baseline to week 28 in airway submucosal inflammatory cells (eosinophils, neutrophils, T cells and mast cells) from bronchoscopic biopsies. Epithelial molecular phenotyping, comprising the three-gene-mean technique, will be used to assess participants’ type 2 (T2) status to enable evaluation of the anti-inflammatory effect of tezepelumab across the continuum of T2 activation. Other exploratory analyses include assessments of the impact of tezepelumab on airway remodelling, including reticular basement membrane thickening and airway epithelial integrity. At the onset of the COVID-19 pandemic, the protocol was amended to address the possibility that site visits would be limited. The amendment allowed for: at-home dosing of study drug by a healthcare professional, extension of the treatment period by up to 6 months so patients are able to attend an onsite visit to undergo the end-of-treatment bronchoscopy, and replacement of final follow-up visits with a virtual or telephone visit.

**Discussion:**

CASCADE aims to determine the mechanisms by which tezepelumab improves clinical asthma outcomes by evaluating the effect of tezepelumab on airway inflammatory cells and remodelling in patients with moderate-to-severe, uncontrolled asthma. An important aspect of this study is the evaluation of the anti-inflammatory effect of tezepelumab across patients with differing levels of eosinophilic and T2 inflammation.

**Trial registration:**

NCT03688074 (ClinicalTrials.gov). Registered 28 September 2018.

## Background

Patients with severe, uncontrolled asthma are at risk of recurrent asthma exacerbations and hospitalizations despite standard-of-care treatment with inhaled corticosteroids (ICS) and long-acting β_2_ agonists (LABAs) [1–3], and consequently experience poor health-related quality of life (HRQoL) [4]. Additional treatment options for these patients include biologic therapies. Current approved biologic therapies for severe asthma target key mediators of type 2 (T2) inflammation in eosinophilic or allergic asthma, including interleukin (IL)-5, IL-4, IL-13 and immunoglobulin (Ig) E, and are prescribed based on indicators of these phenotypes, including dependence on oral corticosteroids (OCS) for disease control [3]. These therapies reduce exacerbations by approximately 50% but do not eliminate them [5–8], and are less effective in patients with lower blood eosinophil counts [9–12]. Furthermore, improvements in physiology and symptom scores are inconsistent [5–8]. Thus, there remains an unmet need for effective therapies, particularly for patients without eosinophilic or allergic phenotypes and those who do not respond to current biologic treatments. A therapeutic approach that has a broader effect on airway inflammation than existing biologics could effectively treat a wide range of patients with severe asthma.

**Fig. 1:**
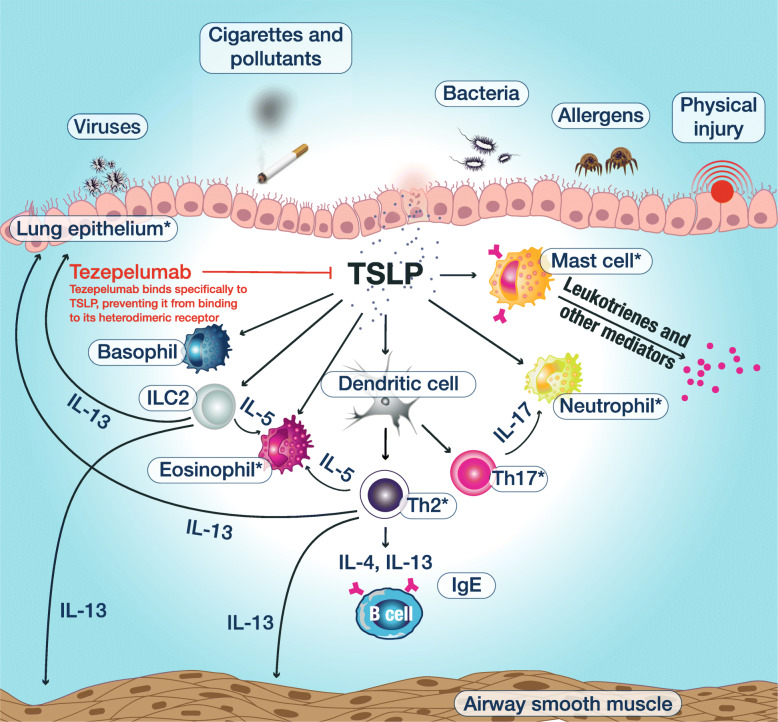
Mechanism of action by which tezepelumab improves clinical outcomes in patients with asthma. TSLP is released from the airway epithelium in response to insults such as virus, allergens and pollutants, triggering an inflammatory cascade. Tezepelumab blocks TSLP from binding to its heterodimeric receptor, thereby inhibiting the production of various inflammatory cytokines and cell types. The primary and secondary objectives of CASCADE comprise assessment of the effects of tezepelumab on the cell types labelled with an asterisk. IgE, immunoglobulin *E; IL*, interleukin; ILC2, group 2 innate lymphoid cell; Th, T helper cell; TSLP, thymic stromal lymphopoietin

Tezepelumab is a human monoclonal antibody (IgG2λ) that binds specifically to thymic stromal lymphopoietin (TSLP), blocking it from interacting with its heterodimeric receptor (Fig. 1) [13–15]. TSLP is an epithelial-derived cytokine implicated in the initiation and persistence of airway inflammation in response to airborne triggers of asthma such as viruses, allergens, pollutants and other airborne irritants that interact with the airway epithelium; it is a key regulator of downstream inflammatory pathways in the airway, both T2 mediated and non-T2 mediated, as well as of other related processes such as epithelial barrier function [16–18]. TSLP has been shown to act in conjunction with other epithelial-derived cytokines, such as IL-25 and IL-33, to promote T2 inflammatory responses [19].

In the phase 2b PATHWAY study, tezepelumab reduced asthma exacerbations by up to 71% compared with placebo in patients with severe, uncontrolled asthma, and improved lung function, asthma control and patient HRQoL [14]. Exacerbation reduction was irrespective of baseline allergic or eosinophilic status, or levels of T2 inflammatory biomarkers [14, 15]. Based on the reduction in exacerbations in a broad population of patients with severe asthma in PATHWAY, including those with low blood eosinophil counts, tezepelumab was granted Breakthrough Therapy Designation by the US Food and Drug Administration in 2019 for patients with severe asthma without an eosinophilic phenotype who are receiving ICS/LABA and additional asthma controllers with or without OCS [20]. The efficacy and safety of tezepelumab in patients with severe, uncontrolled asthma are subsequently being investigated in a programme of ongoing phase 3 trials, including NAVIGATOR (ClinicalTrials.gov identifier: NCT03347279), SOURCE (NCT03406078) and DESTINATION (NCT03706079).

This article describes the design and objectives of CASCADE, an ongoing phase 2 study that is running concurrently with the phase 3 tezepelumab trials noted above. CASCADE aims to explore the mechanism of action of tezepelumab in improving clinical outcomes in patients with moderate-to-severe, uncontrolled asthma across a range of baseline blood eosinophil counts, who are expected to span the spectrum of T2 inflammation. The study will investigate the effects of tezepelumab on airway inflammation and airway remodelling in this patient population, using primarily bronchoscopy-based assessments.

## Methods

### Study design

CASCADE is an ongoing exploratory, phase 2, randomized, double-blind, placebo-controlled, parallel-group study that aims to assess the anti-inflammatory effects of tezepelumab 210 mg administered subcutaneously every 4 weeks (Q4W) for 28 weeks in adults (aged 18–75 years) with uncontrolled, moderate-to-severe asthma (ClinicalTrials.gov identifier: NCT03688074). Patient recruitment for CASCADE has been completed, with a total of 116 patients from 27 sites across five countries randomized (1:1) to receive subcutaneous tezepelumab 210 mg Q4W or placebo Q4W. Patients were stratified by baseline blood eosinophil count as follows: less than 150, 150 to less than 300, and 300 cells/μL or above. To be eligible to participate in the study, patients had to have had moderate-to-severe asthma that was uncontrolled despite treatment with medium- or high-dose ICS plus at least one additional asthma controller medication (e.g. LABAs, leukotriene receptor antagonists, long-acting muscarinic antagonists, cromones or theophylline), with or without maintenance OCS, for at least 3 months before the first study visit. Key additional inclusion and exclusion criteria are shown in Table 1.

**Table 1:** Key inclusion and exclusion criteria

**Key inclusion criteria**	
• Male or female, aged 18–75 years, weight ≥ 40 kg at visit 1	
• Documented physician-diagnosed asthma for ≥12months before visit 1	
• Physician-prescribed asthma controller medication with medium- or high-dose ICS for at least 12 months before visit 1 (as per GINA 2018 guidelines) [21]	
• Documented use of at least one additional maintenance asthma controller medication (e.g. LABA, LTRA, theophylline or LAMA) for at least 3 months before visit 1	
• Predicted normal value for morning prebronchodilator FEV_1_ > 50% and > 1 L at visit 1 or visit 2	
• Documented historical FEV_1_ reversibility of ≥12% and ≥ 200 mL in the 12 months before visit 1 or at visit 2	
• ACQ-6 score ≥ 1.5 at visit 1 or visit 2	
**Key exclusion criteria**	
• Any clinically important pulmonary disease, other than asthma, associated with high peripheral eosinophil counts	
• Any disorder that could, in the opinion of the investigator, affect the safety of the patient or influence the study findings	
• Exacerbation resulting in hospitalization or requiring OCS within 6 weeks of enrolment; more than three asthma exacerbations requiring OCS or hospitalization in the 12 months before visit 1; or exacerbation requiring intubation or admission to the ICU in the year before enrolment	
• Any clinically significant infection requiring antibiotic or antiviral treatment within 2 weeks of visit 1 or during the run-in period	
• Helminth or parasitic infection diagnosed within 6 months of visit 1 that has not been treated with, or is unresponsive to, standard-of-care therapy	
• History of cancer, HIV or hepatitis B or C	
• Current smokers or patients with a smoking history of ≥ 10 pack-years	
• Use of any marked or investigational biologic agent within 4 months or 5 half-lives of visit 1, or any investigational non-biologic agent within 30 days or 5 half-lives of visit 1	
• Use of any immunosuppressive medication within 12 weeks of randomization	
• History of anaphylaxis after biologic therapy	
• Pregnant, breastfeeding or lactating	

*ACQ* Asthma Control Questionnaire, *FEV*_*1*_ Forced expiratory volume in 1 s, *GINA* Global Initiative for Asthma, *HIV* Human immunodeficiency virus, *ICS* Inhaled corticosteroid, *ICU* Intensive care unit, *LABA* Long-acting β_2_ agonist, *LAMA* Long-acting muscarinic antagonist, *LTRA* Leukotriene receptor antagonist, *OCS* Oral corticosteroid

The study aimed to randomize patients, using a capping approach, across a range of baseline eosinophil counts, with approximately 30% having blood eosinophil counts less than 150 cells/μL, 30% having blood eosinophil counts of 150 to less than 300 cells/μL and 40% having blood eosinophil counts of 300 cells/μL or above. The actual distribution of randomized patients across these subgroups is 26, 34 and 40%, respectively.

**Fig. 2:**
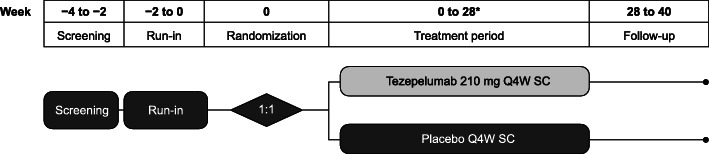
Study design. *For participants who cannot visit the study site at week 28 owing to the COVID-19 pandemic, treatment can be extended to up to 52 weeks until they are able to visit the study site. Q4W, every 4 weeks. SC, subcutaneous

The study was planned to consist of a screening and run-in period of up to 4 weeks, a 28-week treatment period, and a post-treatment follow-up period of 12 weeks (Fig. 2), although the duration of the treatment period may be extended following protocol amendments related to COVID-19 (described below). For the duration of the study, all participants will continue to use their prescribed regimen of maintenance ICS and LABAs without change. The use of short-acting β_2_ agonists as rescue medication is also permitted; however, regular scheduled use of these treatments is not permitted from visit 1 to end of treatment.

Owing to the COVID-19 pandemic that began after the study was underway, the protocol was amended to address the possibility that site visits would be limited. At-home dosing of study drug by a healthcare professional is permitted (if required), and the treatment period can be extended by up to 6 months (with dosing at weeks 28, 32, 36, 40, 44 and 48 as needed) to ensure that participants can continue to receive study drug until circumstances allow them to visit the study site for end of treatment endpoint assessments. This is necessary because bronchoscopies for research purposes are not permitted during the COVID-19 pandemic in the countries in which the study is being conducted. At the onset of the COVID-19 pandemic, approximately 30 out of 116 patients were pending end-of-treatment bronchoscopies; these patients are anticipated to receive prolonged treatment. The amendment also specifies that the final follow-up visit can be replaced by a virtual or telephone visit, which will enable collection of appropriate safety information (and limited efficacy information) in the absence of a final site visit. Two ad hoc meetings were conducted with an external, independent data safety monitoring board, who reviewed the unblinded study data and agreed to the proposed amends to the study protocol. The proposed changes were also approved by health authorities, central institutional review boards and ethics committees for the countries in which the study is being conducted.

Written informed consent was obtained from all patients before enrolment into the study. The study is being conducted in accordance with the principles established in the Declaration of Helsinki and the International Council for Harmonisation Guideline for Good Clinical Practice.

### Objectives and outcome measures

The primary and secondary objectives and endpoints for this study are listed in Table 2. The primary objective of CASCADE is to explore the airway anti-inflammatory effect of tezepelumab compared with placebo over 28 weeks of treatment. This will be measured by the change from baseline in the number of airway submucosal inflammatory cells (eosinophils, neutrophils, T cells and mast cells) in bronchoscopic biopsies at week 28 (or end of treatment if later than week 28) from patients who receive at least 20 weeks of treatment with tezepelumab or placebo, expressed as a ratio. Inflammatory cells and other tissue features will be identified using histology and immunohistochemistry. All histological methods used to explore the primary outcome measure will be pre-validated. The microscope slides generated will be analysed by experts who are blinded to patient treatment and visit number.

**Table 2:** Primary and secondary objectives and endpoints

**Objective**	**Endpoint(s)**^**a**^
*Primary objective*	
To explore the airway anti-inflammatory effect of tezepelumab	Change from baseline to week 28 in airway submucosal inflammatory cells (eosinophils, neutrophils, T cells and mast cells) from bronchoscopic biopsies
*Secondary objectives*	
To explore the effect of tezepelumab on RBM thickening	Change from baseline to week 28 in RBM thickness
To explore the effect of tezepelumab on airway epithelial integrity	Change from baseline to week 28 in airway epithelial integrity
To explore the airway anti-inflammatory effect of tezepelumab across the spectrum of T2 status	Change from baseline to week 28 in airway submucosal inflammatory cells (eosinophils, neutrophils, T cells and mast cells) of bronchoscopic biopsies in patients across the T2 spectrum

^a^Owing to the COVID-19 pandemic, endpoints for some participants may not be assessed at week 28; these participants will receive extended dosing and endpoints will be assessed at timepoints up to week 52

*RBM* Reticular basement membrane, *T2* type 2

As a secondary objective, the primary outcome measures will be explored in patients across a spectrum of T2 inflammation levels. Using RNA sequencing, the three-gene mean [22–24], derived from expression levels of periostin (POSTN), chloride channel accessory 1 (CLCA1) and serpin family B member 2 (SERPINB2) in epithelial RNA isolated from patient bronchial brushings at baseline and end of treatment, will be used to determine the level of T2 inflammation in each individual. Three-gene mean values per patient will be determined by taking the mean of log_2_ transformed transcript per million (TPM) values for POSTN, SERPINB2 and CLCA1, with TPM values less than 0.2 being floored to 0.2, per patient sample. Three-gene mean status will be assessed as a continuum or by quartiles. Additional secondary objectives of CASCADE are to explore the effect of tezepelumab compared with placebo on reticular basement membrane (RBM) thickening and airway epithelial integrity. Change from baseline to week 28 (or end of treatment if later than week 28) in these parameters will be evaluated histologically.

A number of exploratory outcomes will be investigated, including, but not limited to: the pharmacokinetics (PK) of tezepelumab in bronchoalveolar lavage (BAL) fluid and blood; the relationship between inflammatory biomarkers and treatment response to tezepelumab; and the effect of tezepelumab on blood eosinophil and neutrophil counts, and levels of IgE and fractional exhaled nitric oxide (FeNO). Further exploratory outcomes will evaluate the effect of tezepelumab on: airway remodelling in the large airways, assessed using immunohistological markers and computed tomography to estimate airway dimensions and resistance; small-airway obstruction, assessed using impulse oscillometry; airway hyper-responsiveness, measured by mannitol challenge; and lung function, assessed using standard spirometric measures.

Throughout the study, the safety and tolerability of tezepelumab will be evaluated by monitoring: adverse events, serious adverse events and adverse events of special interest; clinical chemistry, haematology and urinalysis; and vital signs and electrocardiograms. An external, independent data safety monitoring board will review the cumulative safety data at regular intervals throughout the study.

### Statistical considerations

All statistical analyses of primary and secondary efficacy outcomes will be performed after database lock, which will occur once patients with delayed bronchoscopies have completed them. Statistical analyses of efficacy outcomes will be performed according to randomized treatment group in patients who complete at least 20 weeks of study treatment (evaluable analysis set), unless stated otherwise. For the primary and secondary endpoints related to airway submucosal inflammatory cells, only patients from the evaluable analysis set with bronchoscopic biopsies at baseline and at week 28 (or end of treatment if later than week 28) will be included in the analyses. Statistical analysis of safety outcomes and the incidence of anti-drug antibodies will be based on all patients, according to actual treatment administered, who receive at least one dose of tezepelumab or placebo. PK analyses will be performed on all patients randomized to the study who receive at least one dose of tezepelumab, irrespective of their protocol adherence and continued participation in the study. Blood and BAL samples that are assumed to be affected by factors such as protocol deviations will be excluded from the PK analyses.

Data on patient demographics and disease characteristics, as well as safety findings, will be reported descriptively for the evaluable analysis set and the full analysis set (all randomized patients who receive at least one dose of study drug irrespective of protocol adherence and continued participation in the study). Changes from baseline to week 28 in the number of airway submucosal inflammatory cells will be expressed as a ratio and evaluated using an analysis of covariance (ANCOVA) model, with blood eosinophil count (< 150, 150 to < 300 and ≥ 300 cells/μL at visit 1), treatment (placebo or tezepelumab) and baseline inflammatory cell count as covariates. The analyses will be performed using log-transformed data, and estimated geometric means and the ratio of geometric means with 90% confidence intervals will be presented. The secondary efficacy endpoints will be analysed using a similar ANCOVA model to that used for the primary endpoint, with additional terms for T2 quartiles and their interactions with treatments. The results for the exploratory endpoints will be summarized using descriptive statistics by treatment group. As CASCADE is an exploratory study, analyses will be conducted without control of type I error and all *p-*values generated will be nominal. Any additional statistical analyses required due to COVID-19 will be pre-specified in the statistical analyses plan before sponsor unblinding and will be described when the results of this study are reported.

With approximately 50 patients per treatment arm and using a 2-sided test with a nominal 10% significance level for each endpoint, it was estimated that the study will have 80% power to observe a reduction in the number of airway submucosal eosinophils if the true effect is a 2.7-fold difference versus placebo, and > 90% power to observe a reduction in the number of airway submucosal neutrophils if the true effect is a 2.7-fold difference versus placebo. A 2.7-fold change was selected for the calculation because it is within the range of effect sizes observed in previous biologic studies [25, 26]. This sample size allows exploratory assessment of the effect of tezepelumab on airway inflammation within quartiles across the T2 continuum and within other subgroups of interest. It is assumed that a small proportion of patients will not have an evaluable primary endpoint value, owing to failed biopsies. To account for this and for dropouts, approximately 55 patients were to be randomized to each treatment arm.

## Discussion

In the phase 2b PATHWAY study, treatment with tezepelumab significantly reduced the annualized asthma exacerbation rate (AAER), irrespective of baseline inflammation status, and improved lung function, asthma control and HRQoL in patients with severe, uncontrolled asthma [14], demonstrating that blocking TSLP can be an effective therapeutic approach in this patient population. The phase 2 CASCADE study is designed to investigate the mechanisms by which tezepelumab exerts an airway anti-inflammatory effect in adults with moderate-to-severe, uncontrolled asthma.

The primary objective of CASCADE is to assess change from baseline in the numbers of multiple airway submucosal inflammatory cells in response to tezepelumab. Previous mechanistic studies of biologic therapies for asthma have focused on counts of airway eosinophils, rather than other inflammatory cell types. Studies of omalizumab have shown that anti-IgE treatment decreases both airway eosinophils and IgE [27–29], while studies of mepolizumab and benralizumab have demonstrated that anti-IL-5 treatment also reduces airway eosinophils [25, 26, 30–33]. Anti-IL-13 treatment with tralokinumab was not effective in reducing bronchial, sputum or blood eosinophils [34]. Results from a mechanistic study of anti-IL-4 treatment with dupilumab have not yet been published, although data posted on ClinicalTrials.gov indicate that it did not meet the primary endpoint of reduction of inflammatory cells in the bronchial submucosa for any of the cell types assessed (tissue eosinophils, CD3+ T cells, CD4+ T cells and chymase- or tryptase-positive mast cells) [35]. For CASCADE, eosinophils, neutrophils, T cells and mast cells were selected as the primary cell types of interest as they characterize airway inflammation in both T2-high and T2-low asthma [36–39]. TSLP has been shown to participate in the activation and/or development of eosinophils [40, 41], neutrophils [42], T cells [43] and mast cells [44–47]. Thus, blocking TSLP with tezepelumab may impact numbers of these cell types in the bronchial submucosa of patients with moderate-to-severe asthma, via effects on cellular activation, development and migration to inflamed tissues, suggesting potential efficacy in a range of patients, including those with non-eosinophilic phenotypes. Furthermore, as TSLP is an upstream regulator of the inflammatory cascade in asthma and influences multiple inflammatory pathways [43, 44, 48, 49], it is anticipated that blocking TSLP with tezepelumab may have wide-ranging anti-inflammatory effects and, therefore, may benefit a broad population of patients with severe asthma who have differing levels of T2 inflammation.

The effectiveness of tezepelumab in a broad patient population has previously been demonstrated in the PATHWAY study, in which exacerbations were improved in response to treatment with tezepelumab in patients with low and high baseline blood eosinophil counts (< 250 and ≥ 250 cells/μL, respectively) and low and high T2 inflammation [14]. To ensure that CASCADE is conducted in a cohort of patients representative of the spectrum of T2 inflammation, the study enrolled patients with a range of baseline blood eosinophil counts (< 150, 150 to < 300 and ≥ 300 cells/μL). Although blood eosinophil count alone may not be sufficient to characterize T2 inflammation fully, for the purposes of this study, it is considered to be an adequate measure for screening patients. After the study is completed, the three-gene mean (bronchial airway epithelial expression levels of *POSTN*, *CLCA1* and *SERPINB2*) will be used to determine the level of T2 inflammation in the participating patients. The three-gene mean has been demonstrated to be suitable as a surrogate marker of T2-driven inflammation in patients with mild-to-moderate asthma [22–24]. This marker has been proposed to correlate with FeNO, blood eosinophils and airway hyper-responsiveness [23], and with clinical markers of T2 inflammation in patients with mild through to severe asthma [50]. We therefore assume that because these tissue-based and clinical markers of inflammation increase in more severe disease, so too will the three-gene mean, which should therefore perform similarly in a population of patients with moderate-to-severe asthma in terms of identifying inflammatory phenotypes and potentially predicting response to therapy. The range of inflammatory phenotypes enrolled in CASCADE is a unique feature of this study compared with previous mechanistic studies of currently approved biologics in asthma, which have been conducted in patients with specific phenotypes [25–33]. Also unique to this mechanistic study compared with previous studies of biologics in asthma is the analysis of tezepelumab PK and inflammatory biomarkers in BAL fluid. Additional exploratory outcomes (e.g. effect on blood eosinophil counts and levels of IgE and FeNO) will be included in CASCADE to investigate and understand further the potential effects of tezepelumab in patients with moderate-to-severe, uncontrolled asthma.

Key learnings from previous mechanistic studies of biologics in asthma were applied to the design of CASCADE, such as ensuring that the study will be of sufficient duration to enable effective evaluation of aspects of airway remodelling. A treatment period of 28 weeks was selected for CASCADE, compared with 12 weeks in previous mechanistic studies such as the MESOS study of tralokinumab, during which airway remodelling was not observed [51]. RBM thickness and airway epithelial integrity were selected as measures of airway remodelling in patients with asthma [52–54] in CASCADE. Given that TSLP may potentially be involved in epithelial homeostasis [55], wound healing [56] and airway smooth muscle proliferation [57], it is expected that RBM thickness and epithelial integrity may be affected by treatment with tezepelumab. Other measures of airway remodelling in CASCADE will include histology and computed tomography to estimate large-airway morphology and resistance, and impulse oscillometry to assess small-airway obstruction. Airway remodelling as a result of biologic treatment has previously been explored in studies of omalizumab, in which changes in RBM thickness were assessed microscopically [58, 59].

CASCADE will use the same dosing regimen (210 mg Q4W) as the ongoing phase 3 trials of tezepelumab in patients with severe asthma. Selection of the 210 mg Q4W dosing regimen was based on efficacy data and exposure–response analyses from the phase 2b PATHWAY study [14, 60]. AAER data from PATHWAY indicated that tezepelumab 210 mg Q4W was more effective than the 70 mg Q4W dosage, whereas the dosage of 280 mg every 2 weeks did not provide additional benefit [14]. Furthermore, based on the primary efficacy endpoint of AAER and the pharmacodynamic endpoint of FeNO, an exposure response to tezepelumab was identified [60]. Tezepelumab was well tolerated at all doses used in PATHWAY (70, 210 and 280 mg) and safety profiles were comparable between the tezepelumab and placebo groups, with no evidence of a dose relationship to treatment-emergent adverse events in the study population [14].

Owing to the COVID-19 pandemic that began after CASCADE was underway, steps were taken to ensure the safety of study participants, to allow adherence to study treatment and to allow study-specific procedures to take place [61]. The protocol was amended to permit at-home dosing of study drug by a healthcare professional and to allow extension of the treatment period by up to 6 months. This ensures that participants can continue to receive study drug until circumstances allow them to visit the study site for end of treatment endpoint assessments. Specifically, bronchoscopies for research purposes are not permitted during the COVID-19 pandemic in the countries in which the study is being conducted. Furthermore, final follow-up visits can be replaced by a virtual or telephone visit. It was important to implement virtual visits and at-home dosing rapidly, because doing so reduced the risk of exposing study participants to SARS-CoV-2. These changes were discussed with local regulatory authorities, ethics committees and institutional review boards before the study protocol was formally amended.

At the onset of the COVID-19 pandemic, enrolment for CASCADE was complete; therefore, recruitment for the study was not affected. However, owing to the extension of the treatment period to allow end of treatment bronchoscopies to take place, database lock is delayed until ongoing patients with delayed bronchoscopies have completed them.

## Conclusion

Patients with severe, uncontrolled asthma are a population with a significant unmet need for new treatments that have wide-ranging effects on airway inflammation and provide greater improvements in asthma outcomes than currently approved biologics and standard-of-care therapies. CASCADE will evaluate the effect of tezepelumab on airway inflammatory cells and airway remodelling in a broad population of patients with moderate-to-severe, uncontrolled asthma and differing levels of T2 inflammation. CASCADE aims to determine the mechanisms by which tezepelumab improves clinical outcomes in patients with moderate-to-severe asthma and to further demonstrate its potential to reduce airway inflammation in patients with eosinophilic or non-eosinophilic asthma.

## Data Availability

Not applicable.

## Abbreviations

AAER: Annualized asthma exacerbation rate
ACQ: Asthma Control Questionnaire
ANCOVA: Analysis of covariance
BAL: Bronchoalveolar lavage
FeNO: Fractional exhaled nitric oxide
FEV_1_: Forced expiratory volume in 1 s
GINA: Global Initiative for Asthma
HIV: Human immunodeficiency virus
HRQoL: Health-related quality of life
ICS: Inhaled corticosteroid
ICU: Intensive care unit
Ig: Immunoglobulin
IL: Interleukin
LABA: Long-acting β_2_ agonist
LAMA: Long-acting muscarinic antagonist
LTRA: Leukotriene receptor antagonist
OCS: Oral corticosteroid
PK: Pharmocokinetics
Q4W: Every 4 weeks
RBM: Reticular basement membrane
RNA: Ribonucleic acid
SC: Subcutaneous
T2: Type 2
TSLP: Thymic stromal lymphopoietin
